# Perceived Effects of Customized Avatars in a Multiplatform Virtual Environment on Pain and Anxiety in Hospitalized Children in Hemato-Oncology

**DOI:** 10.1177/23333936261471667

**Published:** 2026-08-08

**Authors:** Estelle Guingo, Sylvie Le May, Casey Cotes-Turpin, Christine Genest, Sofia Addab, Leandra Desjardins, Pascal Bernier, Michel Duval, Cathy Vézina, Marie-France Langlet, Félix Cotes-Charlebois, David Paquin

**Affiliations:** 1 7001 École des nouveaux médias, Université du Québec en Abitibi-Témiscamingue, Rouyn-Noranda, QC, Canada; 2 25461 Centre de recherche du CHU Sainte-Justine, Montréal, QC, Canada; 3 5622 Facultés des sciences infirmières, Université de Montréal, Montréal, QC, Canada; 4 578596 Centre de recherche de l’institut Universitaire de santé mentale de Montréal, Montréal, QC, Canada; 5 14845 Département en informatique, Université du Québec à Montréal, Montréal, QC, Canada; 6 70357 Centre de recherche de l’hôpital pour enfants Shriners, Montréal, QC, Canada; 7 Centre de Cancérologie Charles-Bruneau, 25461 CHU Sainte Justine, Montréal, QC, Canada

**Keywords:** pain, anxiety, virtual reality, avatar, multiplatform, children, hemato-oncology, Canada

## Abstract

The aim of this pilot study was to explore the feasibility, safety, acceptability, and perceived effects of an immersive, multi-platform distraction (virtual reality/smartphone) intervention on pain and anxiety of children undergoing cancer treatment to improve and refine the intervention. Qualitative data were collected through parent journals and semi-structured, in-person child–parent interviews and analyzed using content analysis to evaluate intervention feasibility and perceived effects on child anxiety and pain management. These data were analyzed through content analysis to evaluate feasibility of the intervention and its perceived effects on the child’s anxiety and pain management. Children’s and parents’ satisfaction were evaluated through surveys. Five children aged from 6 to 17 years hospitalized for cancer treatments and their parents, were recruited (n=10, 5 children, 5 parents). No negative side effects or major logistic issues were reported. Child experiences and parent observations are summarized across three categories: “Effects of the Game Experience,” “Logistical Issues,” and “Engagement and Motivation.” Children and their parents were satisfied with the intervention and mentioned that it had a positive effect on anxiety, pain management, social isolation and children’s mood. Results show the potential of a customized avatar in a multiplatform virtual environment for anxiety and pain management of children hospitalized for cancer treatments. Further research is needed with a larger sample size to have a better understanding of the effects of this intervention with this population of patients.

## Introduction

Hospitalized pediatric hematology-oncology patients are likely to receive anxiety-inducing news regarding their diagnosis, undergo painful treatments, and experience side-effects (Darcy et al., 2014; Karlson et al., 2020; Stone et al., 2018). However, there are few interventions available to help them cope with their day-to-day pain and anxiety and distract them from these symptoms (Lazor et al., 2021; Tutelman et al., 2018).

Digital interventions may be beneficial for pain and anxiety management in children with cancer (Lopez-Rodriguez et al., 2020). Indeed, digital interventions, particularly virtual reality (VR), are suited for pediatric oncology as they provide a non-pharmacological alternative to traditional psychological practices by distracting patients from painful sensory signals and drawing their attention toward immersive, calming virtual experiences. These tools have shown potential to not only reduce procedural pain during invasive treatments like chemotherapy but also to improve emotional well-being by alleviating the anxiety and distress common in hospitalized children (Pittara et al., 2020). A growing body of research has examined the use of virtual reality (VR) as a tool to manage pain and anxiety during painful procedures in pediatric and adolescent populations (Guingo et al., 2026; Hoffman et al., 2020; Osmanlliu et al., 2021) but also for management of anxiety, depression and fatigue (Ioannou et al., 2020). Existing literature suggests that the inclusion of familiar or friendly characters within virtual environments could further mitigate pain and procedure-related anxiety by fostering a sense of security and enhancing emotional engagement (Gold et al., 2021; Han et al., 2019; Liszio et al., 2020). The following review focuses on existing literature regarding the efficacy of virtual reality in managing pain among pediatric and adolescent patients.

VR is a technology simulating an immersive virtual environment through a headset (Ma & Zheng, 2011). During a VR session, users may experience side effects such as nausea, headaches or dizziness (Caserman et al., 2021; Zhang, 2020). As children with cancer have side effects due to their medical treatments, VR content must be carefully designed to avoid adding more. It is important to control VR utilization without limiting engagement in the game. Also, transmedia storytelling is the use of multiple devices to offer diverse ways to interact with the virtual environment. This strategy can be used to ensure long-term game engagement of the patient in VR, while reducing VR play time (Jenkins, 2017). Further, game engagement and motivation may be improved by invoking an emotional response through a non-player character (Hodent, 2017; Isbister, 2016). While few studies have used friendly characters in pain and anxiety management (Chai et al., 2020; Fortier et al., 2016), none offered high-level customization of the visual appearance of the character. The IKEA Effect bias, where individuals assign more value to the objects they build themselves, may contribute to the emotional response invoked by the customization and creation of the avatar (Marsh et al., 2018; Norton et al., 2012).

This led us to conduct a qualitative pilot study to evaluate our multiplatform application (smartphone and VR) using customizable avatars designed by patients themselves to help them better manage their pain and anxiety. By situating the intervention in a real-world environment, we aimed to explore feasibility, safety, acceptability, and perceived effects to improve and refine our intervention. Ultimately, this research will highlight the potential of using child-led design and immersive technologies to create more customized, effective tools for pediatric pain and anxiety management.

## Methods

This pilot study employed a qualitative study design grounded in action research, emphasizing close collaboration among researchers, practitioners, and participants to optimize the development and evaluation of the intervention (MacDonald, 2012). The action-research orientation was chosen to allow iterative feedback loops between clinical team, parents and child with lived-experience and researchers involved, ensuring that the intervention remained clinically relevant and patient-centered. To complement this qualitative approach, descriptive quantitative data were integrated. These were used specifically to assess the technical feasibility of the intervention and the appropriateness of both the clinical outcomes and the data collection tools within the hemato-oncology setting. These measures were not intended for statistical hypothesis testing, but rather to provide objective indicators to guide the iterative refinement of the prototype and inform the design of a future larger-scale study.

### Study Participants and Setting

Convenience sampling techniques were used to recruit pediatric hematology-oncology parent-child dyads (6-17 yo) at a hospital in Montreal, Canada from October 2022 to March 2023. Our multidisciplinary team included healthcare professionals such as an hemato-oncology physician, a head nurse, a computer scientist, a game designer, a 3D artist and a psychologist. To prevent potential bias, team members who had prior clinical relationships with the participants were excluded from recruitment and data collection, ensuring a clear division between clinical care and research activities.

Recruitment was conducted by a research nurse, independent of the primary clinical team, who screened and approached eligible patients. Parents/Child dyads were eligible for study participation if: (1) hospitalisation duration was at least two weeks from the day of recruitment to allow for meaningful familiarization with the technology and ensured that each child could engage with the intervention on multiple occasions, (2) Stable medical condition at the time of recruitment, with no risk of seizure, validated by the healthcare staff or physician, (3) the patient and parents could speak French. Exclusion criteria were: (1) History of seizure, (2) Severe cognitive deficits precluding them to understand the purpose of the study.

### Development of the Intervention

The AVATAR research project was based on a multi-platform game entitled “A Friend for Life” developed by our research team. The intervention itself (a virtual reality game) was developed through a player-centered and co-design approach, allowing experts from diverse fields (child psychology, hemato-oncology, serious games, and human–computer interaction) to iteratively test prototypes and provide feedback to improve usability and therapeutic relevance. A member of the team with lived experience as a pediatric cancer patient also participated in the design process, ensuring that the final experience reflected both professionals’ and patient’s perspectives.

First, the children were invited to draw their own character that were then modeled in 3D and integrated into the game. This game consists of a smartphone application where children could take care of their character, and a VR game where they could play mini games (playable with classic VR hand controllers) along with their virtual friend they created through their own drawing. The scenario duration in VR was set to 10 minutes and the ability to translate into the virtual environment was removed to prevent motion sickness. Children were encouraged to use the application by themselves whenever they experienced pain or anxiety. While the use of VR was primarily patient-initiated, it could also be suggested by parents during peaks of distress or before stressful clinical procedures. Also, they were advised to sit while playing for a better experience and to reduce the risk of motion sickness. Parents could monitor what children were viewing and doing in VR through an external smartphone application (Oculus App). More details and pictures can be found in the conceptual design article (Guingo et al., 2024).

### Data Collection and Measures

Ethical approval for this study was provided by the CHU Sainte-Justine Research Ethics Board [2022-3924] and the Université du Québec en Abitibi-Témiscamingue Research Ethics Board [2022-04]. Written informed consent was obtained from parents, and assent was sought from all participating children.

Children were screened for eligibility using admission lists provided by the hematology–oncology department clerk. Parents and eligible children were approached by a research nurse. After explaining the study to both parent and child, consent and assent were obtained. Each child was then invited to sketch their own virtual friend, which was subsequently modeled in 3D and integrated into the virtual reality (VR) game. Both the VR headset and the companion smartphone application were explained and made available to each child for spontaneous use whenever they felt pain or anxiety during the two-week study period.

Afterwards, parents were given a diary to record their child’s use of the applications, noting pain and anxiety levels before and after each session. Older children had the choice to self-report their anxiety and pain. Anxiety was measured using the 0-4 Children’s Fear Scale (CFS) (McMurtry et al., 2011), a child adapted scale with five faces illustrating an anxiety state (the first one is “not scare” and each next is “a bit more”). Pain was assessed using the 0–10 Numerical Rating Scale (NRS) from 0 (no pain) to 10 (worst pain possible) (Voepel-Lewis et al., 2011). Both scales were explained to parents and children prior to data collection. The diary also included a table to record any observed side effects or technical issues.

Following a two-week period, semi-structured interviews were conducted with both parent and child by a doctoral student trained in qualitative interview techniques. The interview guide included open-ended questions which explored: (1) children’s experiences using the VR game and the app, (2) perceived effects on pain, anxiety, and mood, (3) their feelings toward their personalized avatar, (4) facilitators and barriers from parent’s and child perspectives, and (5) parents’ perceptions of their child’s reactions and the intervention’s usefulness. Prompts were used to encourage elaboration (e.g., “Can you tell me more about that moment?” or “How did you feel when you used the game?”). Examples of questions were: “What do you think about virtual reality?”, “How would you assess the effect of our intervention on the pain experienced by your child?”, “Would you use our prototype again during a future hospitalization?”, “Can you describe [Virtual Avatar’s Name] to me? What do you like and dislike about him/her/it?”, “What improvements would you like to make to the game or avatar?”.

Interviews lasted approximately 30–45 minutes and took place in a quiet hospital room. The semi-structured format was chosen for its flexibility and its ability to elicit detailed, participant-centered narratives aligned with the action-research and content analysis framework (Hsieh & Shannon, 2005).

After the interviews, both parents and children completed a brief satisfaction questionnaire, developed by our team for this pilot study, on their experience using the multiplatform game. Semi-structured interviews were conducted in person with children and their parents from October 2022 to March 2023. Interviews lasted about 35 to 45 minutes and were typically conducted with both the child and parent together. To avoid response mirroring, questions were first directed to the child. When the child was unable to respond or participate, interviews were shortened and conducted solely with the parents.

Due to COVID-19 infection control measures and restrictions, as well as the health condition of patients (cancer patients), headsets could not be shared between participants. Each child received his/her own headset at the beginning of the study, and they could keep it at the completion of the study as an incentive for participation.

### Data Analysis

Data were analysed using a conventional qualitative content analysis approach (Hsieh & Shannon, 2005). This approach enables the systematic classification of textual data through the identification of meaning units, coding, and the development of categories derived inductively from the data. This approach was selected because it allows researchers to integrate experiential, contextual, and emotional dimensions of participant narratives—essential in understanding the lived experience of hospitalized children and their parents during medical recovery.

Recordings from semi-structured interviews were transcribed, anonymized, and analyzed for thematic content identification using the NVivo software. Units of meaning were first identified and coded inductively across transcripts. Codes were then grouped into categories based on similarities and differences in content. Categories were iteratively refined through constant comparison to ensure internal consistency and clear conceptual boundaries. The coding framework was discussed and validated within the research team to enhance analytic rigor and credibility. Representative quotations were translated from French into English for reporting purposes. Measures of pain and anxiety, using respectively the NRS and CFS, were combined and analyzed using descriptive and inferential statistics. Mean values were calculated for pain and anxiety scores before and after the multi-platform intervention. Scores on the Satisfaction survey were combined and analyzed using descriptive statistics.

## Results

### Participants’ Characteristics

Six children and their parents were invited to participate to the study and only one child refused participation. Five children and their parents agreed to participate in the study (4 males 1 female, mean age (SD) = 10.2 years (3.11)) (Table 1). The five participants had different types of cancer or haematologic disease: Acute non-lymphoblastic leukemia (1), acute lymphoblastic leukemia (1), sickle cell anemia (2) and Hodgkin lymphoma (1). While the expected period for the intervention lasted an average of 14 days, in most cases, this period had to be extended due to the health condition of some patients preventing them to participate to the interviews.

**Table 1.** Description of the Child Participants

Characteristics	N=5
Age (years), mean±SD	10.2±3.11
Sex	
Females	1
Males	4
Intervention duration, mean±SD	21.8±5.15
Cause of hospitalisation, n (%)	
Acute non-lymphoblastic leukemia	1
Acute lymphoblastic leukemia	1
Sickle cell anemia	2
Hodkin lymphoma	1

### Children/Parents Experience With the Game

All children participated in interview with one of their parents. Children and parents’ experiences of the virtual reality game (“A Friend for Life”) were captured under three categories: (1) children’s experience and perceived effects of the game, (2) engagement and motivation, and (3) logistic issues and areas for improvement.

#### 1. Children’s Experience and Perceived Effects of the Game

Overall, both children and parents described the intervention as positive and enjoyable. Many perceived the game as reducing pain and anxiety, improving mood, and offering a beneficial escape from the hospital environment. Parents also emphasized emotional and social benefits, particularly through the avatar, which seemed to lessen children’s sense of isolation.

##### Perceived Effects on Pain

When discussing pain, several children and parents reported that the VR experience provided a distraction from discomfort. A 12-year-old explained that “it was great, it made me forget about the pain,” while her parent added that “When she plays, she forgets everything around her. She forgets about her pain, she’s in the game.”. Parents of younger children also observed pain relief, noting that after gameplay, their child “almost forgot about pain” or that “it slightly decreased their pain.” Other participants did not experience pain during the intervention yet still used the game for relaxation or anxiety relief.

##### Perceived Effects on Anxiety

Participants also described anxiety reduction as a key benefit. The oldest child recalled that “there is this one time it really worked,” a perception echoed by his parents, who said that “He was very stressed, and it seemed like he thought, ‘That could really help.’ Indeed, it worked right away.”. Another participant mentioned feeling “less stressed” after playing, while his parent noted no clear change. Parents of younger children frequently reported that the game helped their child relax, especially following medical procedures. Children attributed this effect to the distraction and sense of escape the game provided: “I only think about what I am doing and nothing else,” or “it’s the fact of being outside. To be in another world than what I can see from my hospital window.”. Parents agreed, observing that the game “got him out of his hospital room” and allowed children “to disconnect from the hospital environment.” One mother even noted that her daughter was “completely in her own world. She didn’t know what I was doing or saying.” while playing, showing the depth of immersion.

##### Perceived Effects on Mood and Isolation

Parents consistently mentioned that gameplay improved their child’s general mood, describing their child as “happy,” “motivated,” and “smiling.” One parent mentioned that “when he played, it changed his mood… he’s like exploring a whole new world where he meets his avatar and feels comfortable, home-like”. Children also expressed attachment to their avatar, which some described as a friend. One child said, “it’s like I was talking to someone,” while another added that “the character is friendly.” and another one said, “It was like having a human relationship interaction”. For hospitalized children often confined to their room, these perceived social interactions were important in lessening the emotional weight related to being hospitalized and undergoing painful and stressful treatments.

#### 2. Engagement and Motivation

**Figure 1.**
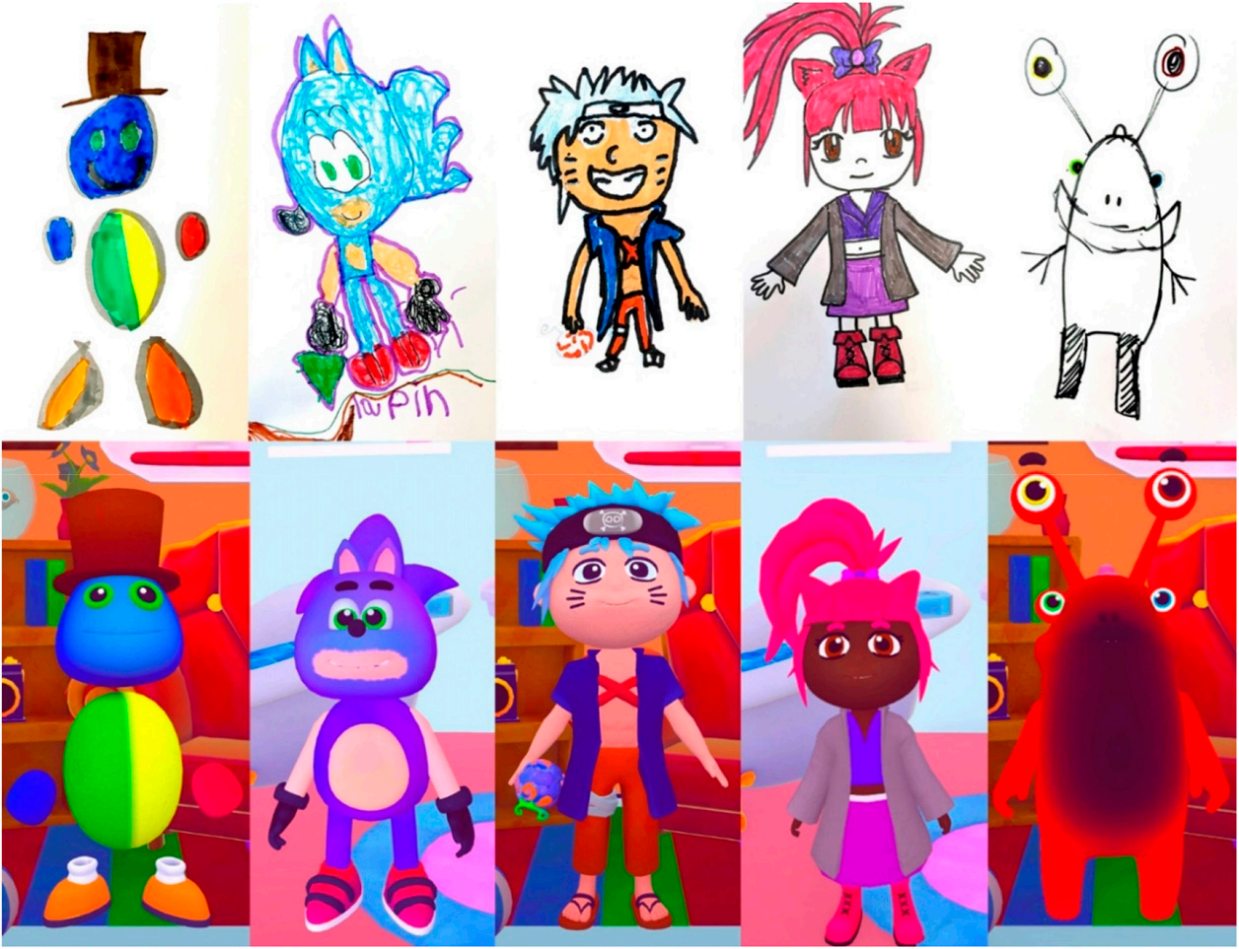
Characters from drawing to 3D

The second category focused on children’s engagement and motivation toward the multiplatform intervention. Parents frequently described their child’s pride in their avatar, their enthusiasm for the game’s mechanics, and, in some cases, their appreciation for the transmedia storytelling that connected the VR and mobile applications (Figure 1).

##### Pride and Ownership

Several parents observed that their child took pride in their own avatar/character, frequently showing it off or talking about it after playing. One parent recalled that her son “was always saying, “Look at my character!’” and another noted that “he was proud… that it was a character he created.”.

##### Avatar’s Appreciation and Transmedia Connection

Children expressed strong attachment to their avatar, describing it as an accurate representation of their drawing. One of them mentioned: “I love everything about him,” while another parent explained that “it looked exactly like the drawing and all the ideas the child initially provided to describe his avatar.” Older children also appreciated the connection between the VR and smartphone applications, commenting that “when I changed it on the smartphone, it changed in the headset too” or that they enjoyed seeing virtual items “appear in the smartphone app.” However, not all participants felt this connection added value. One older child noted that “it was fun but not necessary,” and that he “would have had as much fun using only the VR headset.”

##### Engagement in Gameplay

Parents reported that their child was consistently engaged during and after gameplay. One parent explained that her child “talked about it just after he played or when he discovered something new,” while another shared that her daughter “had so much fun” and “always checked on the phone to see the new features that would appear related to her avatar.” These reflections suggest that the game successfully fostered excitement and sustained engagement across both platforms. Younger children tended to play “for fun,” rather than for anxiety and pain management purposes, leading them to get bored of the game if they played for a longer period. Older children that used the game more sporadically did not indicate any signs of boredom.

#### 3. Logistic Issues and Areas for Improvement

The third category elucidated key logistical challenges and delineated potential areas for improvement in implementing the multiplatform intervention. While technical issues were rare, some parents and children identified aspects related to content, accessibility, and user experience that could be refined in future iterations.

##### Equipment and Technical Issues

Most participants found the technology user-friendly once demonstrated. A few encountered minor issues, such as difficulty streaming the VR experience to the smartphone for parents to observe. As one parent explained, “it’s a great feature, but it didn’t work,” while another said that “once you showed us how it worked, it worked very well.”

##### Content and User Experience

Both parents and children suggested expanding the game’s content to maintain engagement over longer periods. Parents mentioned that “he would have liked to spend more time in the game,” “it was the same thing every day,” and “he got bored of doing the same thing all over again.”. Younger children tend to play more for fun than the older ones, getting bored more quickly. One of the older participants also felt the experience was better suited for younger children. Children offered creative ideas for improvements, such as making the avatar move with them, adding dialogue or mini-games, or even creating a multiplayer platform where hospitalized children could meet and play together. One parent supported this idea, explaining that pre-pandemic, hospitalized children could exchange letters, but a shared digital world could allow social interactions among children by allowing them to meet and interact with each other.

##### Accessibility and Targeted Population

From a usability standpoint, all children were able to play, though younger ones initially needed assistance. As one parent described, “at the beginning I had to stay next to him… now he is more confident, he can handle all of it.” Another noted that “it was easy to learn.” One parent raised an important issue regarding the target population, pointing out that children hospitalized for transplants may not always be able to engage in a digital game due to fatigue, oxygen masks, or acute pain. “Some children had very acute pain… the headset might be too much,” she explained. Parents confirmed that during such times, their child preferred to withdraw from all activity, mentioning “he didn’t want anything, even the TV” or “when he felt pain, he didn’t want to anything but rest.” Still, others used distraction as a coping strategy: “When she was in pain, she liked to distract herself… it helped her forgot about the pain.”

### Pain and Anxiety

Results on pain and anxiety mean scores are shown in Table 2. VR and smartphone application results were combined as parents did not always specify which application was used. We can observe slight encouraging changes in pain and anxiety with some children. Younger children played the most even if they did not feel anxious or in pain.

**Table 2.** Means (SD) on Anxiety and Pain Before and After Game Utilization (Parents’ Journal)

​	ID	Intervention duration (in days)	Number of uses registered in the parent’s journal	Pain maximum score		Mean pain score (SD) (0 to 10)		Anxiety maximum score		Mean anxiety score (SD) (0 to 4)	
				Pre	Post	Pre (SD)	Post (SD)	Pre	Post	Pre (SD)	Post (SD)
​	1	14	22	0	0	0 (0)	0 (0)	0	0	0 (0)	0 (0)
​	2	30	9	0	0	0 (0)	0 (0)	0	0	0 (0)	0 (0)
​	3	22	13	8	6	0.615 (2.22)	0.462 (1.66)	2	1	0.154 (0.55)	0.077 (0.28)
​	4	23	4	5	4	4.25 (0.96)	2 (1.83)	1	0	0.25 (0.5)	0 (0)
​	5	20	3	1	1	1 (0.58)	1 (0.58)	4	1	2 (1.73)	0.33 (0.58)
Total Sample Mean	X	21.8	10.2	2.8	2.2	1.173 (0.75)	0.69 (0.81)	1.4	1.2	0.43 (0.56)	0.08 (0.17)

#### Pain

Two children over five reported changes in their mean pain measure pre (1.173±0.75) and post (0.69±0.81) utilization of the platform (interactions with their avatar). Two of them presented a slight decrease in their pain level. Two children didn’t report feeling any pain.

#### Anxiety

Three children over five reported changes in their mean anxiety measure pre (0.43±0.56) and post (0.08±0.17) utilizations of the platform. All of them presented a slight decrease in their level of anxiety. Two children didn’t report feeling any anxiety.

### Satisfaction

Results on Satisfaction of children and their parents are presented in Tables 3 and 4. Overall, parents were satisfied with anxiety and pain management through the game. All parents strongly agreed to say that the applications were adapted to hospital context and that it didn’t have a negative impact on their child. Children were satisfied with their avatar appearance. One child on five thought that the game didn’t help with anxiety and pain management. Most children (4 of 5) answered the game helped with anxiety management. Both parents and children agreed to say that the games were easy to understand and that they would use the applications again for future hospitalizations.

**Table 3.** Children’s Satisfaction Survey (n=5)

​	No	A bit	Yes
I liked participating in the drawing activity.	0	0	5
I like my avatar’s appearance.	0	0	5
I like the virtual environments of “A Friend for Life”	0	1	4
Explanations allowed me to fully understand how the applications work.	0	1	4
“A Friend for Life” helped me reduce my pain during hospitalization.	1	2	2
“A Friend for Life” helped me to be calmer and to control my emotions during hospitalization.	1	0	4
I liked playing with “A Friend for Life” during hospitalization.	0	0	5
I would like to use “A Friend for Life” again for future hospitalization.	0	0	5

​	Mean score (SD)
On a scale from 0 to 10, what score would you give to “A Friend for Life”?	8.4 (1.52)

**Table 4.** Parents’ Satisfaction Surveys

​	Strongly disagree	Disagree	Agree	Strongly agree	Number of respondants
“A Friend for Life” helped my child to manage their anxiety.	0	0	1	4	5
“A Friend for Life” helped my child to manage their pain.	0	0	2	2	4
The use of “A Friend for Life” had a negative impact on my child’s hospitalization.	5	0	0	0	5
It’s easy to understand how to use the game “A Friend for Life”	0	0	2	3	5
I would use “A Friend for Life” again for future hospitalization.	0	0	1	4	5
The game content was age-appropriate for my child.	0	0	1	3	4
The applications were adapted to hospital context.	0	0	0	5	5
The idea of using a multiplatform game based on customized avatar is an idea that deserve to be developed.	0	0	0	5	5

## Discussion

This study examined children’s and parents’ experiences with our multiplatform intervention combining VR, a mobile application and customized avatars to support pain and anxiety management during hospitalization in hemato-oncology. Overall, participants described the intervention as positive, engaging, and emotionally supportive. All children used the intervention during the trial period for two weeks or more depending on their health status. While patients did not all use the intervention at the same frequency, many of them noticed (or their parent) a positive impact on their anxiety or pain, as reported in parents’ diary and during the interviews. The perceived benefits reported in the interviews are consistent with prior research indicating that immersive virtual reality can reduce procedural pain and anxiety in pediatric patients (Eijlers et al., 2019; Hitching et al., 2023; Pittara et al., 2020; Sánchez-Caballero et al., 2024). The slight improvements observed in pain and anxiety measures for several children further reinforce these impressions.

Both children and their parents were highly satisfied with the game. Also, all parents reported that the intervention did not harm children’s hospitalization journey and was adapted to the hospital context. Participants also discussed logistical and experiential considerations related to the intervention’s use. Overall, only minor technical issues were encountered during the study, and no VR side effects were reported. This underlines the potential feasibility of our multiplatform intervention in the hematology-oncology unit. In their systematic review, Sánchez-Caballero et al. (2024) emphasizes the high satisfaction reported by parents, patients, and healthcare professionals regarding various VR-based interventions for pain and anxiety management. This reinforces the idea that VR is generally well accepted and remains a promising tool to further explore for managing pain and anxiety. Moreover, studies on virtual reality for cancer pain management have focused primarily on acute pain, and its use for everyday pain remains underexplored (Comparcini et al., 2023; Pittara et al., 2020).

Areas of improvement included adapting the game to children’s age were reported. Age targeting is important to consider when creating a virtual experience for pain and anxiety management (Guerra-Armas et al., 2024; Won et al., 2017). Considering these results, offering multiple virtual environments with scalable complexity would allow the intervention to be tailored to the diverse age groups and needs of pediatric patients. The game may be better presented as a possible solution for pain and anxiety management upon request. This way, children may use the headset only for pain and anxiety management and they may not get bored too quickly. That being said, this may bring some challenges as the use of the headset must remain simple for healthcare professionals that are not always familiar with technology (Son et al., 2022). Moreover, character creation require a significant amount of time (48 hours), making it necessary to revise our workflow for larger groups. To streamline the process, one option is to offer children premade avatars with various clothing, accessories, and shape options. While this would reduce the level of customization that was central to the original design, advancements in AI could offer future solutions. For instance, AI could eventually transform children’s drawings into 3D models.

Parents emphasized the need for content expansion to maintain engagement, and both children and parents suggested additional interactive features, such as mini-games, dialogue options, or multiplayer functionalities enabling hospitalized children to connect with one another. These suggestions illustrate the potential for digital interventions to enhance social interactions, complementing their therapeutic benefits. Hasei et al. (2024) tried to explore this area, by offering a metaverse to pediatric, adolescents and young adults’ patients with rare cancer diagnosis to communicate together (n=10). The findings showed that the metaverse allowed patients to interact with peers, exchange personal experiences, and gain emotional support.

While it was not the main goal of the present study, we observed that our intervention may allow children an opportunity for social interactions as it offered them a virtual customized friend to play with. If the relation between user and virtual characters in serious games is not an expanded field, some researchers explored the attachment and the relationship between videogames players and non-player characters (NPCs). Empathy for avatars and non-player characters has been explored through qualitative study (n=10) with experienced videogame players, underlining the existence of virtual empathy (Harth, 2017). There are no studies specifically examining empathy toward NPCs in virtual reality, but one qualitative study highlights the potential for players to experience empathy toward Artificial Intelligence (AI) driven NPCs. (n=25) (Zargham et al., 2025). As our characters were not AI, they didn’t have natural manners and interaction, but it remains an interesting area of improvement. Social isolation in chronically ill patients can lead to anxiety and depression (Clarke & Currie, 2009; Iovino et al., 2023). In hindsight, we think that a social and emotional response optimization of the character with voice recognition and AI could have offered moral support to children.

Furthermore, the game evoked emotions such as pride or happiness and improved the player’s overall mood. This aligns with the findings of Pittara et al. (2020) comprehensive review, which highlights that VR can reduce negative emotions and foster positive mood in patients. All children appreciated their character, and parents noticed their child were proud of the character they created. We think that these feelings could possibly be related to the IKEA Effect (Norton et al., 2012), a cognitive bias linked to the participation in the creation or a construction of an object resulting in a higher valuation of the object. To date, no study has explicitly examined the link between the IKEA Effect and avatar creation. However, within game studies, some research has explored players’ attachment to their self-created avatars (Szolin et al., 2023) as well as their interpretations of the visual aesthetics of non-player characters (Poivet, 2025).

Another important observation concerns the variability in children’s willingness to engage with the intervention depending on their clinical state. Parents noted that during periods of acute pain, fatigue, some children preferred rest and were not inclined to use the headset and remain very passive. Conversely, others actively sought distraction through gameplay during painful episodes. Pain is a common and distressing symptom for pediatric cancer patients, often leading to reduced engagement in normal activities (Klages et al., 2025; Madi et al., 2023). This can be exacerbated by numerous invasive procedures, which are an unavoidable part of cancer treatment and can cause considerable anxiety and pain. These painful experiences can result in an aversion to activity, even activities that would normally be enjoyable. Moreover, a study exploring daily patient-reported outcome measurements show that physical functioning, pain, sleep disturbance, and nausea are among the most common moderate or severe symptoms reported by children with cancer, all of which directly impact their willingness to engage in activities (Meryk et al., 2022). These reflections highlight the need for flexible, child-centered digital tools that accommodate fluctuating medical conditions and individual coping mechanisms.

### Study Limitations

To our knowledge, this study is the first to explore the use of transmedia storytelling and unique customizable avatars. The time-consuming aspect of the avatar conception, the technology shortage at the time of recruitment and the cost of providing individual headsets to participants due to COVID-19 restrictions contributed to a small sample. Nevertheless, given the focused aim of this pilot study, the specificity of the participant group, and drawing on the concept of information power (Malterud et al., 2016), we consider this sample size sufficient to address the aims of this qualitative pilot study. The study demonstrates high information power through its narrow objective, highly specific and homogeneous sample (pediatric hemato-oncology patients), theoretical grounding in action-research, strong quality of dialogue during in-depth interviews, and rigorous content analysis strategy.

Limitations are also due to lack of gender diversity, as only one female participant was included. Consequently, the findings may primarily reflect the experiences of male patients and may not fully capture gender-based nuances in avatar customization or interaction with the VR environment. Future research should aim to explore gender-related influences.

Also, relying solely on participants for data collection may have led to missing data. Also, semi-structured interviews with children can be difficult specifically with younger children that may be shy or unable to provide detailed responses (Banister & Booth, 2005; Kortesluoma et al., 2003; Quintela Do Carmo et al., 2024). Younger children do not always have the capacity to explain or to argue a complex subject such as pain management (Emerson & Bursch, 2020; Jaaniste et al., 2016). Most interviews had to be shortened due to children’s health status, and some participants were distracted by healthcare staff’s usual checkups. Including parent assessments of their child’s pain and anxiety allowed us to collect data throughout the whole study period. However, this poses some limits and bias to the study as a parent that really believed in our intervention may have overestimated the perceived impacts in in their child.

## Conclusion

In conclusion, the use of avatars within a multiplatform virtual environment shows potential benefits for pain and anxiety management in children hospitalized for cancer treatments on a hematology-oncology unit. The applications could help this population by reducing their isolation and improving their general mood. Children were engaged in the intervention. Both parents and children were highly satisfied with the intervention and found that the intervention was useful for pain and anxiety management. Further research is needed to improve our prototype. As well, a larger sample size with a qualitative design might bring more information and set requirements for a future pilot pragmatic randomized clinical trial to provide more data on the feasibility and acceptability of customized digital platform to help hospitalized children managing their symptoms and coping with medical treatments.

## Data Availability

The datasets generated and/or analyzed during the current study are available from the corresponding author on reasonable request.*
